# Supplementary material for: EGFR R521K Polymorphism Is Not a Major Determinant of Clinical Cetuximab Resistance in Head and Neck Cancer
Source: Cancers (Basel). 2022 May 13;14(10):2407. doi: 10.3390/cancers14102407 (PMC9140151; doi:10.3390/cancers14102407)
Supplement: Supplementary file 1 [file cancers-14-02407-s001.zip › cancers-1675164-supplementary.pdf]

cancers

MDPI

Supplementary Materials

EGFR R521K Polymorphism Is Not a Major Determinant of Clinical Cetuximab Resistance in Head and Neck Cancer

Mihály Cserepes, Györgyi A. Nelhűbel, Mónika Meilinger-Dobra, Adrienn Herczeg, Dóra Türk, Zita Hegedűs, Laura Svajda, Erzsébet Rásó, Andrea Ladányi, Kristóf György Csikó, István Kenessey, Árpád Szöőr, György Vereb, Éva Remenár and József Tóvári

1. Methods

1.1. Identification of Genetic Alterations

For the detection of EGFR variant III we used 600 ng total RNA template for reverse transcription by the AMV-RT reverse transcription system (Promega). The produced cDNA served as template for the PCR reacton using PCR primers specific for the regions surrounding the multi-exon deletion site (primer sequences for EGFR vIII: 5′-GCT CTG GAG GAA AAG AAA GGT AAT TAT and 5′- ACG CCG TCT TCC TCC ATC T. Primer sequences for wild-type EGFR detection: 5′ – TAC CTA TGT GCA GAG GAA TTA TGA TCT TT and 5′ – CCA CTG TGT TGA GGG CAA TG). If the specific PCR product was present in the sample, it was cut out from gel, and DNA was purified using NucleoSpin Gel and PCR clean-up kit (Macherey-Nagel), and sent to Microsynth AG for Sanger sequencing.

For the detection of EGFR R521K single nucleotide polymorphism, we used genomic DNA, and the part containing the polymorphic site was multiplied using PCR (primers used for EGFR521 region: 5′ – ACT GCT GTG ACC CAC TCT G and 5′ – TGC CTC GGC TGA CAT TCC). Then, after gel electrophoresis, and purification of specific DNA bands from the gel, the samples were sent for Sanger sequencing, as well. All sequencing data were analyzed, and samples, where EGFR R521K mutant allele had the frequency of 50% or more, were considered as mutant cell lines or mutant clinical tumors.

1.2. Effector Cells Used for ADCC Assay

CD16.176V.NK-92 cells were supplied by Dr. Kerry S. Campbell, (the Fox Chase Cancer Center, Philadelphia, PA on behalf of Brink Biologics, Inc. San Diego, CA), and was generated from the NK-92 cell line (ATCC CRL-2407) derived from a human NK-like phenotype non-Hodgkin’s lymphoma, which lacks endogenous expression of CD16 [1]. This has been transduced to express a high affinity variant (176V) of FcγRIIIA (CD16, see VAR_003960 entry within P08637 and BC017865.1 [2,3]. The cells were cultured in special NK medium of α-MEM containing 10% FCS and 10% horse serum, supplemented with glutamine, non-essential amino acids, Na-pyruvate, antibiotics and IL-2 at 100 IU/ml (Novartis, Basel, Switzerland).
